# Prediction of Skin Sensitization Potential of Silver and Zinc Oxide Nanoparticles Through the Human Cell Line Activation Test

**DOI:** 10.3389/ftox.2021.649666

**Published:** 2021-05-28

**Authors:** Ravi Gautam, SuJeong Yang, Anju Maharjan, JiHun Jo, Manju Acharya, Yong Heo, ChangYul Kim

**Affiliations:** ^1^Department of Occupational Health, College of Bio and Medical Sciences, Daegu Catholic University, Gyeongsan, South Korea; ^2^Department of Toxicity Assessment, The Graduate School of Medical and Health Industry, Daegu Catholic University, Gyeongsan, South Korea

**Keywords:** skin sensitization, nanoparticles, silver, zinc oxide, human cell line activation test (h-CLAT)

## Abstract

The development of nanotechnology has propagated the use of nanoparticles (NPs) in various fields including industry, agriculture, engineering, cosmetics, or medicine. The use of nanoparticles in cosmetics and dermal-based products is increasing owing to their higher surface area and unique physiochemical properties. Silver (Ag) NPs' excellent broad-spectrum antibacterial property and zinc oxide (ZnO) NPs' ability to confer better ultraviolet (UV) protection has led to their maximal use in cosmetics and dermal products. While the consideration for use of nanoparticles is increasing, concerns have been raised regarding their potential negative impacts. Although used in various dermal products, Ag and ZnO NPs' skin sensitization (SS) potential has not been well-investigated using *in vitro* alternative test methods. The human Cell Line Activation Test (h-CLAT) that evaluates the ability of chemicals to upregulate the expression of CD86 and CD54 in THP-1 cell line was used to assess the skin sensitizing potential of these NPs. The h-CLAT assay was conducted following OECD TG 442E. NPs inducing relative fluorescence intensity of CD86 ≥ 150% and/or CD54 ≥ 200% in at least two out of three independent runs were predicted to be positive. Thus, Ag (20, 50, and 80 nm) NPs and ZnO NPs were all predicted to be positive in terms of SS possibility using the h-CLAT prediction model. Although further confirmatory tests addressing other key events (KEs) of SS adverse outcome pathway (AOP) should be carried out, this study gave an insight into the need for cautious use of Ag and ZnO NPs based skincare or dermal products owing to their probable skin sensitizing potency.

## Introduction

The development of nanotechnology has spread the use of nanoparticles (NP) in various fields including industry, agriculture, engineering, cosmetics, or medicine. The cosmetic industry is among the first industry to implement nanotechnology-based materials (Mihranyan et al., 2012). For more than 30 years, nano-based ingredients have been used in the cosmetic industry (Pastrana et al., 2018; Carrouel et al., 2020). Nanomaterials based topical medicines or cosmetics render special benefits over micro-scale materials. The higher surface area and unique physiochemical properties of nanoparticles lead to higher transport of ingredients through the skin (Ahmad et al., 2018; Fytianos et al., 2020). The key goals of using nanoparticles in skincare products are controlled release of ingredients, increased efficacy, occlusive properties, physical stability, or active transport of targeting (Kaul et al., 2018).

It is claimed that among various nanoparticles used in consumer products, silver nanoparticles (Ag NPs) hold the highest degree of commercialization (Henig, 2007) and Ag NPs are incorporated in 30% of products that contain nanomaterials (Wijnhoven et al., 2009). Ag NPs' excellent broad-spectrum antibacterial properties and minimal side effects have led their use in detergents, bandages, catheters, antibacterial sprays, shoes, food storage containers, clothing, water disinfectants, etc (Vigneshwaran et al., 2007; Crosera et al., 2009; Augustine et al., 2016; Khan et al., 2018). AgNPs are applied in nanocomposites, anti-caries formulations, implant coatings, treatment of oral cancer and local anesthesia in dentistry (Noronha et al., 2017). Furthermore, AgNPs' role in cancer diagnosis, cancer treatment, cardiovascular implants, orthopedic and orthodontic implants and fixations, and as antimalarial agents is increasing their use in the medical field as well (Murphy et al., 2015; Rai et al., 2017; Huy et al., 2019). Owing to their useful properties, zinc oxide (ZnO) nanostructures have attracted a great deal of interest for novel applications in cosmetics, pigments and coatings, biomedical imaging, drug delivery, antibacterial agents, catalysts, diabetic treatment, wound healing, anti-cancer or anti inflammatory agent (Zhang et al., 2013; Shi et al., 2014; Kim S. et al., 2017; Mishra et al., 2017). ZnO NPs have also been used to enhance flame retardancy and thermal stability in textile fabrics, as well as moisture management, thermal insulation, electrical conductivity, and hydrophobicity (Verbič et al., 2019). ZnO NPs render significant bactericidal properties and are thus used as an antimicrobial agent in food industries (Seil and Webster, 2012; Sirelkhatim et al., 2015). Also, ZnO NPs are consistently used in sunscreens to confer better ultraviolet (UV) protection and to make products transparent and aesthetically acceptable compared to their larger opaque counterparts (Cross et al., 2007; Wang and Tooley, 2011). According to a survey in 2012, ZnO was used in 235 domestic cosmetic products of Korea with concentrations of 0.05–17% and powder type products were highest among the cosmetic containing ZnO (Kim K. B. et al., 2017).

While consideration in the use of nanoparticles is increasing, concerns have been raised regarding their potential impact on human health. The larger surface area of NPs offers larger numbers of atoms or molecules leading to higher reactivity. ZnO NPs showed lower TC-50 (concentration inducing 50% cell mortality) in THP-1 cells than its micron-sized and also showed higher toxicity in human T cells (Prach et al., 2013; Sahu et al., 2016). Similarly, another study screening the toxicity of nano and micro sized silver in human hepatocyte cell lines depicted that AgNP showed significantly higher toxicity than micro-Ag (Liu et al., 2011). Regarding the dermal exposure of nanoparticles, it has been previously reported that metallic NPs can pass through the stratum corneum, hair follicles, and sebaceous glands (Rancan et al., 2012). ZnO nanoparticles penetrated the stratum corneum of sunburned pig and hairless mice and led to collagen loss in rats applied with sunscreen containing 20 nm ZnO for 28 days (Wu et al., 2009; Osmond and McCall, 2010; Monteiro-Riviere et al., 2011; Surekha et al., 2012). In studies, sunscreen containing ZnO NPs and microparticles applied to human subjects resulted in a higher measurable concentration of zinc in blood and urine of the subjects who were exposed to ZnO NP-containing products than the product with ZnO microparticles (Gulson et al., 2010, 2012). Similarly, Ag-NPs are reported to penetrate intact skin with increased permeation through damaged skin (Larese et al., 2009). Since ZnO and Ag NPs penetrate the skin, their interaction with skin proteins and immune cells could potentially lead to cutaneous immune reactions including skin sensitization (Grundström and Borrebaeck, 2019). Although various assessments related to ZnO and Ag NPs' toxicities have been performed, skin sensitizing potential remains unexplored.

Skin sensitization (SS) consists of a type IV hypersensitivity response triggered after repeated dermal exposure to a possible allergenic substance in susceptible individuals. Every manufactured commercial product must be evaluated for its skin sensitizing potential, however, the use of animals for testing skin sensitizing potential has been prohibited by European Union (EU) regulations (European Commission, 2006; Chung et al., 2018). Various alternative test methods have been adopted by the Organization for Economic Co-operation and Development (OECD) to measure if a chemical ingredient causes a key event (KE) on the adverse outcome pathway (AOP) for SS. According to the OECD, the AOP for SS has been divided into four KEs: interaction of chemicals with skin proteins and formation of the hapten-protein complex (KE-1 or molecular initiating event, MIE), inflammatory keratinocytes response (KE-2), activation of dendritic cells (KE-3) and activation and proliferation of T-lymphocytes (KE-4) (OECD, 2014). Among various OECD adopted methods, TG 422E deals with the *in vitro* skin sensitizations assays addressing the key events on activation of dendritic cells on the AOP for SS. It consists of the human Cell Line Activation Test (h-CLAT), the U937 cell line activation test, and the interleukin-18 reporter gene assay (OECD, 2018). Of these three methods, h-CLAT evaluates the ability of chemicals to upregulate the expression of dendritic cell activation markers (CD86 and CD54) in THP-1 cells, a human monocytic leukemia cell line. The h-CLAT method has been shown to possess 85% accuracy (*n* = 142) in distinguishing skin sensitizers from non-sensitizers with a sensitivity of 93% and specificity of 66% compared to local lymph node assay (LLNA) results (OECD, 2018). Moreover, this method has been successfully employed to predict the skin sensitizing potential of biocides: polyhexamethylene guanidine (PHMG) and triclosan (TCS) with or without excipient propylene glycol (PG) producing consistent results as obtained using local lymph node assay: 5-bromo-2-deoxyuridine-flow cytometry method (LLNA: BrdU-FCM) (Joo et al., 2019; Yang et al., 2021). This method has also been employed to screen the skin sensitizing potential of biodegradable polymers, carbon nanohorns (CNHs), and hydroxyethylcellulose (HEC) nanofibers (Jung et al., 2011; Selvam et al., 2020).

Different *in vitro* assays to differentiate between skin sensitizers and non-sensitizers have been carried out. A study investigated single-walled carbon nanotubes, titanium dioxide, and fullerene nanomaterials using mDPRA and U-SENS^TM^ and categorized them as skin sensitizers through combined results of the assays (Bezerra et al., 2021). Using OECD adopted, the ARE-Nrf2 Luciferase KeratinoSens^TM^, representing the second key event of AOP for SS, nanoparticles such as copper oxide (CuO), cobalt monoxide (CoO), cobalt oxide (Co_3_O_4_), nickel oxide (NiO), and titanium oxide (TiO_2_) were tested to classify CuO and CoO as skin sensitizers (Kim et al., 2021b). Similarly, other studies using the same method predicted CuO as sensitizer and carbon nanotubules as non-sensitizer (Kim et al., 2020, 2021a). Yoshioka et al. highlighted the indirect mechanism of skin sensitization by metal nanoparticles where nanoparticles presented metal ions to lymph nodes and induction of metal ion-specific CD4^+^ T cells and production of IL-17 led to skin sensitization or allergic response (Yoshioka et al., 2017). Activated dendritic cells play a crucial role in priming specific T cell response and h-CLAT assay addresses the process of activation of dendritic cells following exposure to sensitization. Data on the use of h-CLAT to screen the skin sensitizing potential of nanoparticles is little and successful application of this method could aid on discrimination of NP's skin sensitizing property. This present study used the h-CLAT to predict the skin sensitizing potential of ZnO and Ag NPs, which are consistently used in the commercial products that are exposed to skin. Although there are restrictions on testing insoluble substances using an *in vitro* alternative test methods for prediction of skin sensitization, few insoluble materials and nanoparticles have been evaluated by KeratinoSens™ assay or ARE-Nrf2 Luciferase-Keratinosens™ assay (Andres et al., 2013; Settivari et al., 2015; Kim et al., 2020, 2021a). Furthermore, OECD TG 422E recommends using a stable suspension of substances with lower or no solubility. Thus, this study could present an insight into whether the use of nanoparticles in commercial dermal or skincare products is rational or precautions need to be taken.

## Materials and Methods

### Test Chemicals and Preparation of NP Suspension

Eight substances were used for the proficiency test for the h-CLAT described in OECD TG442E Appendix II (OECD, 2018) as follows: nickel sulfate (CASRN 10101-97-0), 2-mercaptobenzothiazole (CASRN 149-30-4), R(+)-Limonene (CASRN 5989-27-5), imidazolidinyl urea (CASRN 39236-46-9), isopropanol (CASRN 67-63-0), glycerol (CASRN 56-81-5), lactic acid (CASRN 50-21-5), 4-aminobenzoic acid (CASRN 150-13-0). All the chemicals were purchased from Sigma-Aldrich (St. Louis, Missouri, USA) except nickel sulfate (Alfa Aesar, Ward hill, Massachusetts, USA). All the chemicals were coded and provided to the experimenter. The experimenter had no idea about the chemical's identifications. 2,4-dinitrochlorobenzene (DNCB, CASRN 97-00-7) was used as a positive control in every assay carried out. Two independent experiments were performed for the proficiency test chemicals since the two runs were concordant on defining as positive or negative, and therefore a third run was not necessary.

Zinc Oxide powder (water dispersion, 20 weight%; size: 30~40 nm) was purchased from US Research Nanomaterials, Inc (Houston, TX, USA) and 20, 50, and 80 nm silver nanosphere (silver purity 99.99%, 1.03, 1.08, and 1.04 mg/ml respectively in aqueous 2 mM citrate) were purchased from nanoComposix (San Diego, CA, USA).

The suspension of NPs in media were prepared as described by Kim et al. with few modifications (Kim et al., 2021a). Saline was used as a vehicle for the dispersion of nanoparticles. Stock solutions prepared in saline were sonicated at 40 kHz with 100 W output power for 10 min in a bath-type sonicator (KODO, Gyeonggi-do, Korea). Then, the stock solutions were diluted to different working concentrations (1:50 dilution) using RPMI supplemented with 10% FBS followed by short sonication for 2 min. The dispersion of NPs in media was checked under the microscope and no further characterization was carried out.

### Cell Culture

The cell culture procedures generally followed the ECVAM Good Cell Culture Guidelines and the techniques have been already addressed in the previous report (Hartung et al., 2002; Coecke et al., 2005; Lee et al., 2019). The THP-1 cell line was purchased from American Type Culture Collection (Manassas, VA, USA) and cultured in RPMI-1640 with L-glutamine and 25 mM HEPES (Gibco, Waltham, MA, USA) supplemented with 10% fetal bovine serum (Gibco), 0.05 mM 2-mercaptoethanol (Sigma-Aldrich) and 1% penicillin-streptomycin mixture (Gibco). The cells were considered suitable for h-CLAT assay as their doubling time was 38 h which is within the range (30–55 h) defined in DB-ALM Protocol n°158 and the reactivity check with DNCB, nickel sulfate, and DMSO provided expected results, as detailed in the OECD TG 442E (OECD, 2018) and the DB-ALM Protocol n°158: human Cell Line Activation Test (h-CLAT) (EURL-ECVAM, 2016). In this study, the relative fluorescence intensity (RFI, %) for CD86 and CD54 with 4 μg/ml DNCB were 611 and 426%; 200 μg/ml nickel sulfate were 290 and 2,195%, and 2,000 μg/ml lactic acid were 117 and 138% respectively. The cell viability of (0.2%) DMSO or media vehicle control was 95–98%.

### h-CLAT Assay

All the procedures followed the OECD TG 422E and DB-ALM protocol n°158. First, dose-finding assays were performed. In this assay, the concentration that maintained 75% cell viability (CV75) compared to the vehicle control (culture media) was calculated. For this, THP-1 cells (10^6^ cells) were cultured with 1 ml of various concentrations of nanoparticles in 24 well plate and incubated for 24 h at 37°C in a 5% CO_2_ incubator. Eight different two-fold diluted concentrations of nanoparticles were used. Concentrations ranged from 1,000 to 7.8 μg/ml for ZnO NPs and concentrations for 20, 50, and 80 nm Ag NPs ranged from 20.8 to 0.163 μg/ml, 21.6 to 0.169 μg/ml and 20.8 to 0.163 μg/ml, respectively. Two independent runs were carried out to determine the average CV75. Cell viability was determined by fluorescence-activated cell sorting (FACS) analysis (BD ACCURI^TM^, BD Bioscience, San Jose, CA, USA) using propidium iodide (PI, Sigma-Aldrich) staining at the concentration of 0.25 μg/FACS tube.

To derive a single prediction of whether a substance is positive in terms of surface markers expression, three independent experiments for NPs were performed and the h-CLAT test method prediction model was applied. For the experiments, THP-1 cells (10^6^) were incubated with eight serial concentrations (1.2 × CV75, CV75, 1/1.2 × CV75, 1/1.2^2^ × CV75, 1/1.2^3^ × CV75, 1/1.2^4^ × CV75, 1/1.2^5^ × CV75, 1/1.2^6^ × CV75). DNCB (4 μg/ml) was used as positive control and 0.2% DMSO was used as its vehicle control. After 24 h of incubation, cells were washed twice with FACS buffer followed by blocking with FACS buffer containing 0.01% globulin solution (Cohn fraction II, III human, Sigma-Aldrich) at 4°C for 15 min. Then cells were split into three aliquots in round-bottom polystyrene tubes and stained with FITC labeled anti-CD86 (BD-Pharmingen), anti-CD54 (Dako, Denmark), or mouse IgG1 isotype control (Dako) antibodies at 4°C for 30 min. After washing two times with FACS buffer, cells were suspended in FACS buffer and PI was added before cell acquisition by flow cytometer. The Relative fluorescence intensity of CD86 and CD54 was calculated based on mean fluorescence intensity (MFI). An additional experiment to rule out the possibility of fluorescence interference due to nanoparticles was carried out, for this, nanoparticles at working concentrations in the absence of cells were checked for their FITC fluorescence compared to non-stained cells.

### Decision of Skin Sensitizing Positivity

In h-CLAT, a substance was predicted as positive if, in at least 2 of 3 independent experiments, any tested concentrations yielded CD86 or CD54 RFIs of ≥150%, or ≥200% (CV ≥ 50%), respectively. After a substance was predicted to be positive, the effective concentration (EC) for CD86 and/ or CD54 expression was calculated as described in the OECD TG 442E. The EC150 for CD86 and the EC200 for CD54 are the concentrations at which the test substance induced an RFI of 150 or 200%, respectively.

## Results

### Proficiency Test and Cell Viability

The proficiency test was performed to demonstrate the technical proficiency of the h-CLAT before its routine use. All eight tested substances were correctly categorized as positive or negative by h-CLAT. Nickel sulfate, 2-mercaptobenzothiazole, R(+)-limonene, and imidazolidinyl urea were correctly classified as positive while isopropanol, glycerol, lactic acid, and 4-aminobenzoic acid were classified as negative (Table 1). The proficiency variables of four sensitizers (CV75, EC150, EC200) fell into the reference ranges provided in OECD TG 422E. Even though according to OECD TG 422E, CD86 RFI value for 2-mercaptobenzothiazole and R(+)-limonene is negative, here both the chemicals demonstrated CD86 RFI more than 150%. Moreover, EC values are within the reported reference range in case of positive results. CV75s were determined for all four NPs. No cell toxicity was observed for 20, 50 nm, and 80 nm Ag NPs at tested concentrations and the highest treated concentrations were considered to be CV75 of the respective NPs. In the case of ZnO, 25.8 μg/ml was determined as the average CV75 of two independent assays (Table 2).

**Table 1:** Proficiency test results of eight recommended substance in the OECD TG 442E for h-CLAT assay.

**Test substances^**a**^**	***In vivo* prediction**	**CV75 reference range (μg/ml)**	**TG 442E results for CD86 (EC 150 reference range in μg/ml)**	**TG 442E results for CD54 (EC200 reference range in μg/ml)**	**Results (1st run, 2nd run) and prediction**			
					**CV75 (μg/ml)^**b**^**	**CD86 RFI (%) (EC150, μg/ml)^**c,d**^**	**CD54 RFI (%) (EC200, μg/ml)^**c,d**^**	**+ Prediction (CD86 ≥ 150% and/or CD54 ≥ 200%)**
Nickel sulfate	S (moderate)	30–500	+(<100)	+(10–100)	218, 162	371, 262 (46)	2,423, 1,829 (51)	+
2-Mercaptobenzothiazole	S (moderate)	30–400	–(>10)^e^	+(10–140)	166, 160	510, 233 (85)	526, 752 (59)	+
R(+)-Limonene	S (weak)	>20	–(>5)^e^	+(<250)	180, 376	144, 473 (239)	254, 1,012 (121)	+
Imidazolidinyl urea	S (weak)	25–100	+(20-90)	+(20-75)	37, 42	462, 214 (42)	690, 463 (31)	+
Isopropanol	NS	>5,000	–(>5,000)	–(>5,000)	>1,000	97, 114 (N/A)	153, 176 (N/A)	-
Glycerol	NS	>5,000	–(>5,000)	–(>5,000)	>1,000	139, 72 (N/A)	132,122 (N/A)	-
Lactic acid	NS	1,500-5,000	–(>5,000)	–(>5,000)	>1,000	127, 92 (N/A)	133, 181 (N/A)	-
4-Aminobenzoic acid	NS	>1,000	–(>1,000)	–(>1,000)	>1,000	88, 102 (N/A)	100, 197 (N/A)	-

*S, sensitizer; NS, non-sensitizer, N/A, not applicable; EC, effective concentration; RFI, relative fluorescence intensity; h-CLAT, human Cell Line Activation Test; CV75, 75% cell viability*.

a *The test substances were coded and distributed to experimenters before the h-CLAT assays*.

b *Two independent runs were performed to determine the CV75 concentrations*.

c *The two highest RFI% of two independent runs among the eight serially diluted concentrations that were tested*.

d *The EC150 for CD86 or the EC200 for CD54 is the average concentration from two independent runs at which the test substance induced an RFI of 150% or 200%, respectively*.

e *Historically, a majority of negative results have been obtained for this marker and therefore a negative result is mostly expected. The range provided was defined on the basis of the few historical positive results observed*.

**Table 2:** Prediction of the SS potency of silver and zinc oxide nanoparticles by h-CLAT assay.

**Nanoparticles**	**Size**	**Test run**	**CV75 (μg/ml)^**a**^**	**CD86 RFI (%) (EC150, μg/ml)^**b**^**	**CD54 RFI (%) (EC200, μg/ml)^**b**^**	**+ SS prediction = CD86 ≥ 150% and/or CD54 ≥ 200%**	**Final prediction on sensitization (positive if two of three runs are +)**
Silver	20 nm	1st	20.6	161 (17)	326 (11)	+	Positive
		2nd		174 (11)	285 (8)	+	
		3rd		109 (N/A)	248 (17)	+	
Silver	50 nm	1st	21.6	146 (N/A)	1,205 (8)	+	Positive
		2nd		109 (N/A)	169 (N/A)	-	
		3rd		181 (8)	880 (6)	+	
Silver	80 nm	1st	20.8	260 (8.0)	10,006 (8)	+	Positive
		2nd		156 (25)	234 (11)	+	
		3rd		249 (8)	4,381 (8)	+	
Zinc Oxide	30~40 nm	1st	25.8	217 (12)	3,922 (10)	+	Positive
		2nd		263 (13)	4,622 (11)	+	
		3rd		360 (9)	4,721 (11)	+	

*SS, Skin Sensitizer; RFI, relative fluorescence intensity; h-CLAT, human Cell Line Activation Test; CV75, 75% cell viability; EC, effective concentration; N/A, not applicable*.

a *Average CV75 of two independent runs*.

b *The highest RFI % among the eight serially diluted concentrations that were tested*.

### Prediction of the Skin Sensitizing Potential of Silver and Zinc Oxide Nanoparticles

**Figure 1:**
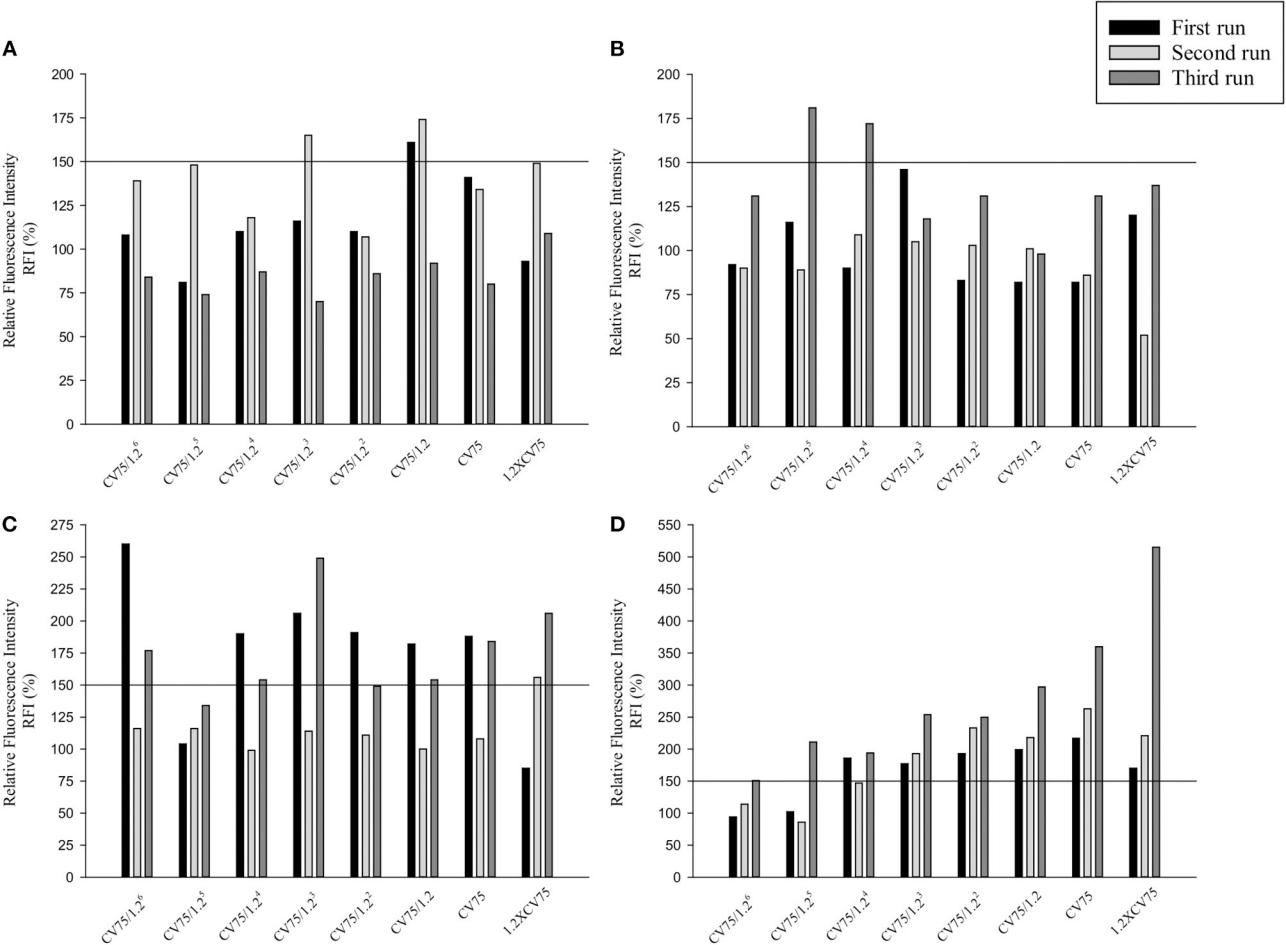
Relative Fluorescence Intensity (RFI) data of three different runs for CD86 of **(A)** 20 nm AgNPs **(B)** 50 nm AgNPs **(C)** 80 nm AgNps and **(D)** ZnO NPs at different concentrations (1/1.2^6^ × CV75, 1/1.2^5^ × CV75, 1/1.2^4^ × CV75, 1/1.2^3^ × CV75, 1/1.2^2^ × CV75, 1/1.2 × CV75, CV75, 1.2 × CV75). RFI were obtained by calculating the percentage of CD86 Mean Fluorescence Intensity (MFI) of NPs treated cells compared to CD86 MFI of solvent/vehicle treated cells after reduction of CD86 MFI of isotypes of respective concentrations.

**Figure 2:**
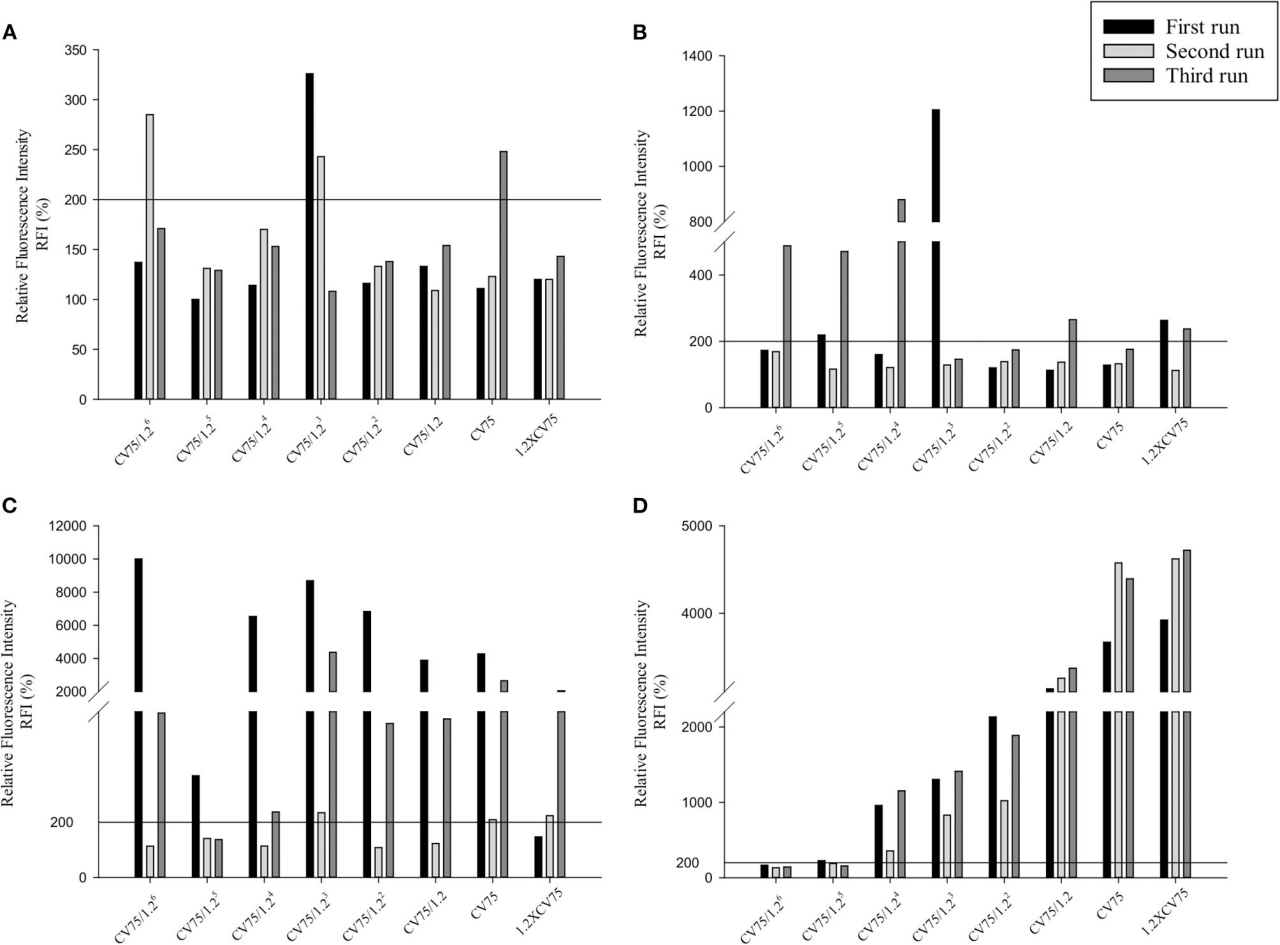
Relative Fluorescence Intensity (RFI) data of three different runs for CD54 of **(A)** 20 nm AgNPs **(B)** 50 nm AgNPs **(C)** 80 nm AgNps and **(D)** ZnO NPs at different concentrations (1/1.2^6^ × CV75, 1/1.2^5^ × CV75, 1/1.2^4^ × CV75, 1/1.2^3^ × CV75, 1/1.2^2^ × CV75, 1/1.2 × CV75, CV75, 1.2 × CV75). RFI were obtained by calculating the percentage of CD54 MFI of NPs treated cells compared to CD54 MFI of solvent/vehicle treated cells after reduction of CD54 MFI of isotypes of respective concentrations.

Three independent runs were performed to screen 20, 50, and 80 nm Ag and ZnO NPs. According to the OECD TG 422E, h-CLAT predicts that a substance is positive if in two runs the CD86 RFI is ≥150% and/or the CD54 RFI is ≥200% (with CV ≥ 50%) at any tested concentration (OECD, 2018). All the acceptance criteria were met for the h-CLAT assay, RFI for CD86 and CD54 with 4 μg/ml DNCB were >150 and >200% respectively with average cell viability of 80–86%. The average cell viability of (0.2%) DMSO and media ranged from 95 to 98% and the cell viability for all the tested concentration of NPs were higher than 50%. All CD86 RFI (Figure 1) and CD54 RFI (Figure 2) of different concentration of NPs are considered to be actual RFI of CD86 and CD54 expressed by THP-1 cells and not due to the interference of nanoparticles as each CD86 RFI and CD54 RFI was obtained after reducing the MFI of isotype for their respective concentrations. Moreover, the λmax for ZnO NPs, 20 nm Ag NPs, 50, and 80 nm NPs were 335, 391, 424, and 455 nm, according to the manufacturer's information and do not coincide with FITC fluorescence. Moreover, nanoparticles did not give FITC fluorescence when tested in absence of cells and antibody staining. All four nanoparticles were predicted to be positive with only 50 nm silver classified as negative in one run (Table 2). The final average EC value was calculated using EC150 or EC200 values of positive tests among three independent tests. EC150 and EC200 of 20 nm Ag were 14 and 12 μg/ml, of 50 nm Ag were 8 and 7 μg/ml, of 80 nm Ag were 14 and 9 μg/ml and of zinc oxide were 11 and 11 μg/ml.

## Discussion

Increasing use of nanoparticles in cosmetics or dermal-based products has escalated nanoparticle exposure in the workforce or customers. Despite their tremendous potential benefits, little is understood about the short-term or long-term health effects. Concerns have been raised about the potential risks that could result from the skin penetration of nanoparticles after application in the form of cosmetics or other dermal products. Skin sensitization is a key endpoint for the safety evaluation of chemicals in cosmetics and personal care products (Settivari et al., 2017). However, the skin sensitization potential of nanoparticles used in various consumer products is the least studied. Thus, in this study, we employed the h-CLAT assay to predict the skin sensitization potential of ZnO and Ag NPs. ZnO and Ag NPs independent of their size were predicted to be skin sensitizers based on the h-CLAT prediction model outcomes. Induction of cell membrane markers, CD86 and CD54, in this study is believed to be due to NPs' intrinsic property and not related to the dispersion vehicles. ZnO NPs were dispersed in water and AgNPs were dispersed in 2 mM sodium citrate which was further diluted at least 50 times to prepare working concentrations resulting in quite low concentrations which could rarely cause a change in expression of markers. In concordance to our consideration, in a study, occlusive application of 10% aqueous sodium citrate solution for 20 min did not result in any immediate reactions or non-immunologic contact urticaria (Lahti, 2000).

Traditionally, micro-scale ZnO has been used in sunscreens owing to its ability to filter UVA and UVB radiations. Meanwhile, manufactures have shifted from micro formulation to nanoformulations as nano-sized ZnO improved the transparency and viscosity of sunscreens. The presence of ZnO in sunscreens makes skin a major route of exposure. Although ZnO was previously categorized as a non-toxic and non-skin irritant, size-dependent differential toxicity between micro and nano-scale materials has been observed (Patnaik, 2003; Franklin et al., 2007; Karlsson et al., 2009). It is further reported that ZnO nanoparticles were retained in the stratum corneum and accumulated in hair follicle roots or skin folds in humans (Zvyagin et al., 2008). According to studies, the release of metal ions during the dissolution of nanomaterials could cause toxicity; and cytotoxicity of metal oxides such as ZnO and CuO NP is most likely due to their water-soluble ions (Cho et al., 2012; Jeong et al., 2018). Despite the fact that ions do not easily pass through cell membranes, toxic intracellular concentrations are achieved through a “Trojan horse” mechanism in which metal ions are released from NPs that cross the cell membrane (Cho et al., 2011; Hsiao et al., 2015). Cytotoxicity, oxidative stress due to ZnO is thought to be due to internalization and solubilization of ZnO inside the cell leading to increased intracellular [Zn2+] levels, which disrupts the Zn-dependent enzymes and transcription factors (Pandurangan and Kim, 2015). Nano-sized ZnO demonstrated a higher potential to induce toxicity and inflammation than micro-sized ZnO in THP-1 cells. Proinflammatory cytokines such as IL-1β, IL-6, and TNF-α and inflammatory markers like intracellular adhesion molecule (ICAM)-1, IL-8, and monocytes chemoattractant protein(MCP)-1 were induced by ZnO nanopowder with size <100 nm (Gojova et al., 2007; Sahu et al., 2014). One of the studies done with THP-1 macrophage showed that ZnO nanoparticles were able to activate similar pathways as viruses, they induced PAMP dependent pathways (TLR, RLR), cytokines (IFNs, TNF), and inflammasome secreting IL-1β (Poon et al., 2017). In primary human epidermal keratinocytes, ZnO internalized and induced cytotoxicity and genotoxicity, the range of cytotoxic dose ranged between 8 and 20 μg/ml (Sharma et al., 2011). In another study using THP-1 and MTT assay, ZnO NPs with a spherical diameter of 53.6 nm showed a much lower IC25 value of 2.33 and 5.54 μg/ml in two different participating laboratories however in our study the CV75 was higher than demonstrated in the previous studies. This could be due to the differences in the techniques used for the estimation of cell viability. ROS generation and induction of proinflammatory cytokines are linked to the skin sensitization potential of a chemical (Haas et al., 1992; Esser et al., 2012). Although ZnO nanopowder has been seen to induce both ROS generation and pro-inflammatory cytokines upregulations, thorough investigation on its skin sensitizing potential is lacking. The present study for the first time evaluated the skin sensitization potency of ZnO nanopowder using h-CLAT. ZnO was classified as positive by h-CLAT assay since RFI for both CD86 and CD54 was higher than 150 and 200, respectively in all three runs.

Silver is considered a natural biocide. Ag NPs have shown high antimicrobial efficacy against bacteria, viruses, and other eukaryotic microorganisms and have been enormously used in consumer products such as deodorizing sprays, facial creams, clothing used for preventing body odors, baby wipes, etc (Gong et al., 2007). Ag NPs could be exposed to humans via dermal route during manufacturing or use of the products. Ag NPs in lower but detectable amounts can penetrate the intact skin and the absorption is increased in case of damaged skin, this makes consumers or manufacturers more vulnerable to skin-related diseases including skin sensitization. AgNPs toxicity, like ZnO, could be attributed in part to high local intracellular concentrations of silver ions added to cells through the “Trojan Horse” mechanism (Park et al., 2010; Gliga et al., 2014; Helmlinger et al., 2016). Ag NPs have been studied for their toxicity in various cell lines and found to induce cytotoxicity and genotoxicity. Ag NPs damaged mitochondria and increased ROS causing DNA damage in normal lung fibroblasts (Asharani et al., 2008). AgNPs induced higher expression of IL-1β and TNF- α at any tested concentration compared to its bulk counterpart AgNO_3_ including higher expression of cell surface markers such as ICAM-1, CD86, and IL-8 receptor alpha (CXCR1) in THP-1 which are the signs of DC activation after the encounter to a skin sensitizer (Poon et al., 2017; OECD, 2018). Also, Ag NPs induced cytotoxicity dose-dependently and increased inflammatory proteins IL-1β, IL-6, IL-8, and TNF-α in human epidermal keratinocytes (Samberg et al., 2010). In addition to dose-dependent toxicity, Ag NPs' uptake into cells is believed to be size-dependent. In THP-1 cells, Ag NPs uptake was in order 20>50=75 nm in culture media without fetal calf serum (FCS) and 50=75>20 nm in the presence of FCS (Kettler et al., 2016). In another study, HeLa cells favored intermediate size with uptake in the following order: 50>30>74>14>100 on NPs number basis (Chithrani et al., 2006). In this study, independent of particle size, 20, 50, and 80 nm Ag NPs were classified as positive according to the h-CLAT prediction. Among three, 50 nm Ag NP was positive in two of three runs while the other two were positive in all three runs.

A few previous studies have classified topical application of Ag and ZnO NPs to be relatively safer regarding their skin toxicity. Application of agglomerated ZnO NPs in human volunteers for 5 days did not cause any local toxicity in viable epidermis owing to their inability to penetrate below skin furrows (Mohammed et al., 2019). Similarly, a study using various *in vivo* techniques concluded that ZnO NP was relatively safe as it did not induce acute dermal toxicity, dermal irritation and corrosion, and skin sensitization (Kim et al., 2016). Furthermore, Ag NPs ranging from 7 to 20 nm in a gel formulation for topical application showed no sign of dermal toxicity in Sprague-Dawley rats (Jain et al., 2009). Our findings contradict these previous observations, as all the nanoparticles were positive in terms of expression of surface markers associated with the process of activation of monocytes and dendritic cells following exposure to sensitizers. Our method speculated these nanoparticles having the potential to cause skin sensitization however this method of assessing skin sensitizing potential addresses the third key event (KE3) of AOP for SS. Although h-CLAT assay has been adopted by the OECD, the method is accountable only after a particle encounters the dendritic cells irrespective of its skin penetration ability. OECD No. 256 sets out twelve separate defined approaches (DA) of Integrated Research Strategies (ITS) for the identification and classification of skin sensitizers using *in silico* and *in vitro* techniques based on four skin sensitization AOP KEs, such as “2 out of 3” and sequential testing strategies based on KEs1-3 and KE1 and 3. These approaches allow for a broad evaluation of exposure, skin penetration, metabolism, and key events (OECD, 2016). Further confirmatory assays addressing other KEs of AOP for SS need to be carried out to conclude them as potential skin sensitizers. Although the permeability of NPs through real skin is debatable and the stratum corneum acts as a barrier for penetration, the possibility of permeability of NPs from dermal products below the stratum corneum cannot be disregarded. The application of NPs containing dermal product in the sweating condition is supposed to facilitate the dissolution and increase solubilized metals below stratum corneum due to lower pH of the skin and also due to high prevalence of skin disease in the global population, sunscreen, cosmetics or dermal products could be applied to impaired skin because of an individual's skin condition or potentially because of prior damage of skin by environmental factors (Hay et al., 2014; Seth et al., 2017; Yoshioka et al., 2017; Holmes et al., 2020). Thus, findings from this study suggest the limited use of ZnO and Ag-NPs in dermal products to avoid probable skin sensitization. Moreover, the use of NPs' based products on damaged or fractured skin could lead to higher penetration of the skin by NPs causing a higher risk of skin sensitization.

## Data Availability Statement

The original contributions presented in the study are included in the article/supplementary material, further inquiries can be directed to the corresponding author/s.

## Author Contributions

All authors listed have made a substantial, direct and intellectual contribution to the work, and approved it for publication.

## Conflict of Interest

The authors declare that the research was conducted in the absence of any commercial or financial relationships that could be construed as a potential conflict of interest.
